# How Volatile Compounds, Oxidative Profile and Sensory Evaluation Can Change with Vacuum Aging in Donkey Meat

**DOI:** 10.3390/ani10112126

**Published:** 2020-11-16

**Authors:** Aristide Maggiolino, José Manuel Lorenzo, Gerardo Centoducati, Rubén Domínguez, Francesca Rita Dinardo, Rosaria Marino, Antonella della Malva, Andrea Bragaglio, Pasquale De Palo

**Affiliations:** 1 Department of Veterinary Medicine, University of Bari A. Moro, 70010 Valenzano, Bari, Italy; aristide.maggiolino@uniba.it (A.M.); francesca.dinardo@uniba.it (F.R.D.); andrea.bragaglio@uniba.it (A.B.); pasquale.depalo@uniba.it (P.D.P.); 2 Centro Tecnológico de la Carne de Galicia, Parque Tecnológico de Galicia, 32900 Ourense, Spain; jmlorenzo@ceteca.net (J.M.L.); rubendominguez@ceteca.net (R.D.); 3 Área Tecnología de los Alimentos, Facultad Ciencias de Ourense, Universidad de Vigo, 32004 Ourense, Spain; 4 Department of Agricultural Food and Environmental Sciences, University of Foggia, 71121 Foggia, Italy; rosaria.marino@unifg.it (R.M.); antonella.dellamalva@unifg.it (A.d.M.)

**Keywords:** donkey meat, volatile compounds, oxidative profile, sensory evaluation

## Abstract

**Simple Summary:**

Aging in donkey meat was never investigated. It represents an important process, because it leads the muscle to become meat. There are many ways to age meat, and vacuum aging is one of these. The present paper characterised donkey meat Volatile Organic Compounds (VOCs) production during 14 vacuum aging days, its oxidative status and the consequent sensory evaluation. Lipid oxidative processes are delayed, but some protein oxidative processes happen, influencing VOCs production and sensory evaluation.

**Abstract:**

This study aims to improve knowledge on donkey meat and the vacuum aging effect on the Volatile Organic Compounds (VOCs), oxidative profile and status and the sensory characteristics. Ten 18-month old Martina Franca donkeys’ male foals were involved in the trial. *Longissimus thoracis* (LT) muscle was extracted from each left half carcass, between the fourth and the ninth rib. Each muscle was divided into five sections, vacuum packaged, stored at 2 °C, and randomly assigned to one of the different aging time (1, 3, 6, 9, and 14 days of aging). Volatile compounds, oxidation parameters, and antioxidant enzymes were analysed, and a sensory test was performed. A nested one-way analysis of variance (ANOVA) was performed for aging time as an independent variable. Significance was set at *p* < 0.05. Aldehydes are the most produced VOCs, but no changes were observed during vacuum aging (*p* > 0.05). Nitrogen compounds increased during aging (*p* < 0.01). TBARs and hydroperoxides did not change during the storage, whereas the protein carbonyls increased (*p* < 0.05). Vacuum aging slowed down lipid oxidation and put in evidence the presence of protein oxidation and degradation, influencing the VOCs productions and sensory evaluation.

## 1. Introduction

Donkeys (*Equus asinus*) were domesticated in Northeast Africa thousands of years ago [1] and today are still working animals that contribute to million people life in developing countries. In Europe and specifically in Italy, donkeys have been employed as working animals until the Second World War; however, with the improvement of the mechanization in agriculture and with the mechanization and the emptying of rural areas in favour of urban ones, the importance and the number of donkeys dramatically decreased [2]. Donkeys are perceived as a land protective, eco-friendly, and innovative activity; moreover, they contribute to the rural development. Actually, in the European Union, donkey farming has another appeal. Particularly, donkey rearing is now considered to have a renewed interest mainly for milk [3,4,5] and meat production [2,6,7].

The acceptance of donkey meat, likewise horse, have been conditioned by the productive destiny of the species. Although there are traditional dishes of asinine fresh meat, mainly in Northern Italy, nevertheless several foods, e.g., dry cured sausages, were originally obtained from animals that were slaughtered at the end of working live [6,8]. It is known that the flesh of equids is rejected, especially in the English-speaking world [9,10]; in fact, its consumption appears virtually absent in the United Kingdom, the United States and Australia [11]. Actually, agriculture, meat and milk productions, social activities, and also tourism and leisure represented the principal types of uses of donkey in Europe [12]. Donkey meat is particularly consumed in some southern European countries, such as Spain and Italy, where, although there is not a great accuracy in data collecting, it is well known that is used for some typical dishes. It has been recognized as a nutritive food for human consumption with good-quality proteins, vitamins, and minerals, low intramuscular fat content, and great iron concentration [7]. Despite that the meat of equids has been perceived too as second-choice food over the past years (including in horse-consuming nations), its appeal is deeply evolved [6,13,14,15,16,17]. Consumer acceptability of meat can be influenced by many aspects, such as nutritional properties, tenderness and texture characteristics, colour, and marbling, particularly linked to visual appearance, and mostly sensory profile [18,19,20]. All of these characteristics can be influenced by many factors and aging, applied in meat industry for years, had great implications on consumer acceptability [18]. Meat sensory acceptability may depend on cultural factors, themselves linked to regional customs, particularly for donkey meat. Although it has been extensively studied in different species, focusing on improving meat flavour, sensory profile, and consumer perception, few studies focused on equids meat aging and its effects on sensory and aroma characteristics, and the most of them on horse meat [18,21,22,23,24,25], and no one on donkey meat. There is a lack of knowledge on aging technique and their effects on donkey meat characteristics and consumers’ perceiving. Flavour and sensory profile are key factors that are able to determine the consumer satisfaction and, consequently, the potential meat market [26], and reactions, such as sugar reductions, lipid oxidation, and hydrolysis, are the principal mechanisms that are linked to aroma compounds formation during storage and cooking [27]. On the other hand, aging is the way that leads the muscle transformation in meat, improving eating quality and leading to the release of substances (free amino acids, peptides, free fatty acids) themselves substrate for flavour compounds formation [28]. Thousands of volatile compounds have been identified in cooked meat, some of them being able to affect sensory attributes and consumer perception of the meat, but no studies were conducted until now on donkey meat.

This study aims to start to fill the gap of knowledge on donkey meat and the vacuum aging effect on the Volatile Organic Compounds (VOCs), oxidative profile and status, and the sensory characteristics.

## 2. Materials and Methods

Ten Martina Franca donkeys’ male foals were involved in the trial. They were born from March to May 2017 and fattened until slaughter age (18 months) in the same farm. They were individually reared and fed with the same rations and feed supply.

At the age of 18 months, each animal was transported and slaughtered at a European Community-approved abattoir, in compliance with European Community laws on Animal Welfare in transport (1/2005EC) and the European Community regulation on Animal Welfare for slaughter of commercial animals (1099/2009EC) located at 22 km from the farm. The journey time was about 20 min. After slaughtering, carcasses were dressed following commercial dressing-out procedures at the abattoir [29]. No electrical stimulation was used. Immediately after slaughter, carcasses were chilled at 4 °C in a chilling room for 24 h. Afterwards, *Longissimus thoracis* (LT) muscle was extracted from each left half carcass, between the fourth and the ninth rib. Each muscle was divided into five sections, vacuum packaged, stored at 2 °C, and then randomly assigned to one of the different aging time (1, 3, 6, 9, and 14 days of aging); cranial and caudal sections were randomized across aging time. For packaging the Besser Vacuum^®^ film (Besser Vacuum, Dignano, Udine, Italy) was used. It is characterised by 65 µm thickness, 63 g/m^2^ of weight, ≤65 cm^3^/m^2^ × day × bar of oxygen permeability at 23 °C and 0% of relative humidity and ≤3.5 g/m^2^ × day of water vapor permeability at 23 °C and 85% of relative humidity. Ten samples were obtained and analysed at each aging time.

### 2.1. Chemical Composition

Chemical composition was performed exclusively on samples at first aging day. Moisture [30], protein [31], intramuscular fat [32], and ash [33] content were calculated according the International Organization for Standardization (ISO) methods. The samples were analysed in triplicate.

### 2.2. Volatile Compound Analysis

All of the procedures were performed at each aging time. Samples (5 g) of foal steaks were grilled at 130–150 °C for 5 min. on each surface, using an electrical griddle (Delonghi, Mod. CG660, Treviso, Italy). A heating treatment was considered complete when all the steaks reached an internal temperature of 70 °C measured with a copper constantin fine-wire thermocouple fixed in the geometrical centre of the sample (Model 5SCTT-T-30-36; Omega Engineering Inc., Norwalk, CT, USA). After cooking and cooling, the samples were minced while using a commercial grinder (Moulinex/Swan Holding Ltd., Birmingham, UK), vacuum-packed, and stored at −30 °C for no longer than two weeks until analysis.

#### 2.2.1. SPME Extraction

The SPME tool from autosampler was loaded with a fused-silica fibre (10 mm length) coated with a 50/30 mm thickness of DVB/CAR/PDMS (divinylbenzene/carboxen/polydimethylsiloxane) (Supelco, Bellefonte, PA, USA). Before the analysis, the fibre was conditioned by heating in a SPME Fibre Conditioning Station at 270 °C for 30 min. For headspace SPME (HS-SPME) extraction, 1 ± 0.02 g of each sample was weighed in a 20 mL vial (Agilent Technologies, Santa Clara, CA, USA) and subsequently screw-capped with a laminated Teflon-rubber disc. The extractions were carried out at 37 °C for 30 min., after equilibration of the samples for 15 min. at the same temperature, ensuring a homogeneous temperature for sample and headspace.

#### 2.2.2. Chromatographic Conditions

After the extraction procedure, the fibre was transferred to the injection port of the gas chromatograph–mass spectrometer (GC-MS) system (7890B GC-System; Agilent Technologies, Santa Clara, CA, USA and a mass selective detector 5977B MSD; Agilent Technologies, Santa Clara, CA, USA). The column that was used for volatile separation was a DB-624 capillary column (30 m, 250 µm i.d., 1.4 μm film thickness; J&W Scientific, Folsom, CA, USA). The chromatographic conditions and mass spectrometer parameters were previously described [34].

#### 2.2.3. Data Processing

After chromatographic analysis, all of the data were analysed with the software Mass Hunter Quantitative Analysis B.07.01. A new method from acquired scan data with library search was created. The integration was done with Agile2 algorithm, while peak detection was done with deconvolution. Compounds were identified by comparing their mass spectra with those that were contained in the NIST14 library (National Institute of Standards and Technology, Gaithersburg, MD, USA). The compounds were considered to be correctly identified when their spectra presented a library match factor >85%. After integration, peak detection, and identification of each compound, the Extraction Ion Chromatogram (EIC) from the Quantifier Ion was obtained from each peak. The results were expressed as area units of the EIC × 10^3^ per gram of sample (AU-EIC × 10^3^/g of sample).

### 2.3. Thiobarbituric Acid Reactive Substances (TBARS), Hydroperoxides and Protein Carbonyls Analyses

Raw minced samples (5 g) were placed in a 50-mL test tube and homogenized with 15 mL deionized distilled water (DDW). Homogenate (1 mL) was transferred to a glass tube for the TBARS determination and 0.05 mL of butylated hydroxytoluene (7.2% in ethanol) was added along with 1.950 mL of thiobarbituric acid (TBA)/trichloroacetic acid (TCA)/HCl (0.375% TBA, 15% TCA, and 0.25 N HCl). The sample solution was shaken and then incubated at 90 °C for 15 min. in a thermostatic bath. After this period, the samples were cooled to room temperature (15–30 °C) and then centrifuged at 2000× *g* for 15 min. Supernatant absorbance at 531 nm was measured against a blank containing 2 mL of TBA/TCA/HCl solution in 1 mL of distilled water. The TBARS were calculated when comparing with a standard curve constructed with 1,1,3,3-tetramethoxypropane, and the concentration of lipid oxidation was expressed as milligrams of malondialdehyde (MDA) per kg of meat [35].

For hydroperoxides quantification, 2 mL of homogenate (previously prepared for the TBARS determination) were added with 4 mL of CH_3_OH and 2 mL of CHCl_3_. The samples were vortexed for 30 s and added with 2 mL of CHCl_3_ and 1.6 mL of 0.9% NaCl. The samples were shaken for 1 min. and then centrifuged at 3500× *g* for 10 min. at 4 °C. Two millilitres of lipid extract were sampled from the lower chloroform phase and then processed with 1 mL of CH_3_COOH/CHCl_3_ and 50 μL of KI (1.2 g/L mL distilled water). Samples were stored for 5 min. in a dark room and added with 3 mL of 0.5% of CH_3_COOCd and then vortexed and centrifuged at 4500× *g* for 10 min. at 40 °C. Absorbance at 353 nm was measured against a blank tube in which meat homogenate was replaced by 2 mL of distilled water [36]. The results were expressed in micromoles per gram according to Buege and Aust [35].

Meat samples (2 g) were homogenized in 20 mL of 0.15 M KCl for 2 min. Two aliquots of homogenate (50 μL each) were added with 1 mL 10% TCA and then centrifuged at 1200× *g* for 3 min. at 4 °C to measure protein oxidation. The first aliquot was used as a standard and added with 1 mL of 2 M HCl solution. The second aliquot was added with 1 mL of 2 M HCl containing 10 mM 2,4-dinitrophenyl hydrazine (DNPH). The samples were incubated for 1 h at room temperature (15 to 30 °C) and shaken every 20 min., and then 1 mL of 10% TCA was added. The samples were vortexed for 30 s and centrifuged three times at 1200× *g* for 3 min. at 4 °C and the supernatant removed. Care was taken not to disrupt the pellet. The pellet was washed with 1 mL of ethanol:ethyl acetate (1:1), shaken, and centrifuged three times at 1200× *g* for 3 min. at 4 °C and the supernatant removed. The pellet was then dissolved in 1 mL 20 mM sodium phosphate 6 M guanidine hydrochloride buffer. The samples were then shaken and centrifuged at 1200× *g* for 3 min. at 4 °C. Carbonyl concentration was calculated on the DNPH treated sample at 360 nm with a Beckman Coulter DU800 (Beckman Instruments Inc., Brea, CA, USA) and expressed as nanomoles carbonyl per milligrams protein. Protein concentration was calculated according to the Biuret assay [37,38].

### 2.4. Superoxide Dismutase, Catalase and Glutathione Peroxidase Activity Evaluation

Two samples of 400 mg of raw meat were homogenized in a tissue homogenizer 4 mL saline at 4 °C. The homogenate was centrifuged at 4 °C for 20 min. at 7000× *g* and the supernatant was collected to determine the antioxidant enzyme activities. Plasma was analysed as it was.

Superoxide dismutase (SOD, EC 1.15.1.1) was evaluated by Misra [39] method. The activity was determined from its ability to inhibit the autoxidation of epinephrine. The stimulation of epinephrine autoxidation by traces of heavy metals present as contaminants in the reagents or by the other metals under investigation was prevented by adding 10–4 M EDTA in the buffer in order to chelate these ions. One unit of SOD is defined as the amount of enzyme required to inhibit the rate of epinephrine autoxidation by 50%. The enzyme activity was expressed as U/mg protein.

Catalase (CAT, EC 1.11.1.6) activity was assayed by the method of [40], by following the decrease in absorbance of H_2_O_2_ at 240 nm (e = 40 M^−1^ cm^−1^). One unit of enzyme activity is defined as the amount of enzyme that is required to degrade 1 micromole of H_2_O_2_ in 1 min. and it is expressed as U/mg protein.

Glutathione peroxidase (GPx, EC1.11.1.9.) activity was measured by method of [41]. The reaction measured the rate of GSH oxidation by tert-butyl hydroperoxide, catalyzed by GPx. GSH was maintained at constant concentration by the addition of exogenous GR and NADPH, which converted the GSSG to GSH. The rate of GSSG formation was then measured by the change in the absorbance of NADPH at 340 nm (e = 6.2 mM^−1^ cm^−1^) and activity expressed as nanomoles of NADPH oxidized/min/mg protein.

### 2.5. Sensory Analysis

Sensory analysis was performed by an eight-person trained taste panel. The panels were selected for their sensory acuity using the British Standards Institution (BSI, 1993) methods. At each experimental time, samples were unpackaged and, after sampling five grams for VOC’s analysis and 5 g for enzymes and oxidative profile, were cooked as previously described for VOCs. Connective tissue and fat were trimmed, and the muscle cut into about 2 cm^3^ blocks, which were wrapped in pre-labelled foils and placed in a heated incubator until given to the assessors. The samples were tasted in an order based on the designs outlined by MacFie, et al. [42] for balancing the carryover effects between samples. The panel test was organized in four different sitting sessions for each panellist, at each aging time. During two sessions, each panellist received four samples for each session and, during the other two sessions, each panellist received six samples for each sessions, for a total of 20 samples for each panellist (two samples for each one of the ten donkeys) at each experimental aging time. The samples were randomised by the sensory panel software, in a different order for each panellist. Tested samples were scored on a 1–10 point scale for tenderness (1 = extremely tough to 10 = extremely tender), juiciness (1 = extremely dry to 10 = extremely juicy), overall licking (1 = extremely disliking to 10 = extremely licking), sweetness, unpleasant taste, meaty odour, and unpleasant odour, (1 = extremely weak to 10 = extremely strong).

### 2.6. Statistical Analysis

Data were tested for normal distribution and variance homogeneity by Shapiro–Wilk test. After were subjected to a nested one-way analysis of variance (ANOVA) while using the SAS program. The independent variable was the aging time (1, 3, 6, 9, and 14 days). The mean values and standard error of the means (SEM) were calculated. When a significant effect (*p* < 0.05) was detected, means were compared while using the Tukey’s test.

## 3. Results

Donkey meat chemical composition showed mean moistures values of 73.04 ± 1.58 g/100 g of meat, mean protein values of 18.06 ± 0.83 g/100 g of meat, mean intramuscular fat values of 1.52 ± 0.52, and mean ash values of 1.06 ± 0.19 g/100 g of meat (data not shown).

122 Volatile Organic Compounds (VOCs) have been identified from donkey (*Equus asinus*) *Longissimus thoracis* muscle during the aging process. Table 1 shows the effect of aging time in the aromatic hydrocarbons (12 VOCs) and aldehydes (18 VOCs) of donkey meat steaks aged for 14 days under vacuum conditions. The total amount of aromatic hydrocarbons and the most abundant furan, 2-pentyl- showed decreased values at the sixth day of ageing compared to the 1st (*p* < 0.01).

The total amount of aldehydes did not show significant differences (*p* > 0.05), although some singular and poor represented aldehydes showed few statistical differences with prro increasing trend during aging as Propanal,2-methyl-, butanal butanal, 3-methyl-, benzaldehyde, benzeneacetaldehyde, and 5-ethylcyclopent-1-enecarboxaldehyde (*p* < 0.01).

Table 2 summarizes the effect of aging time in the (linear) hydrocarbons (50 VOCs) of donkey meat steaks that were aged for 14 days under vacuum conditions. The total amount of hydrocarbons did not show significant differences (*p* > 0.05), although only pentane and the 2-octene, (E)- showed poor variation during ageing (*p* < 0.01).

Table 3 shows the effect of aging time in the ketones (16 VOCs) content of donkey meat steaks aged for 14 days under vacuum conditions. The total amount of ketones did not show significant differences (*p* > 0.05). Only 2,3-pentanedione, 2-hexanone and 3-octanone, 2-methyl-, poorly representing in the total amount, showed significative variation during aging (*p* < 0.01).

The effect of aging time in Alcohols (12 VOCs), carboxylic acids (3 VOCs), nitrogen compound (five VOCs), and sulphur compound (2 VOCs) content of donkey meat steaks aged for 14 days under vacuum conditions are reported in Table 4. Ageing did not affect the alcohols production (*p* > 0.05); differently, the total carboxylic acids and total nitrogen compounds were significantly affected by aging time (*p* < 0.01) showing an increasing trend. Finally, the aging time did not affect the sulphur compounds (*p* > 0.05).

The effect of aging time in TBARs, hydroperoxides, protein carbonyls, superoxide dismutase, catalase, and glutathione peroxidase values of donkey meat steaks aged for 14 days under vacuum conditions are reported in Table 5. TBARs values at nine and 14 aging days are higher than those observed at three aging days (*p* < 0.01). Differently, hydroperoxides and protein carbonyls did not show any variation during the storage time (*p* > 0.01). Moreover, all of the antioxidant enzymes displayed an increase day by day during the aging time (*p* < 0.01).

Table 6 presents the effect of aging time in sensory properties of of donkey meat steaks aged for 14 days under vacuum conditions. Sweetness, meaty odor, and overall liking tend to increase during the aging time with higher values at 14 aging days compared to one day (*p* < 0.05). However, the other sensorial parameters did not show any significant difference due to aging time (*p* > 0.05). 

## 4. Discussion

There is a great variation in volatile compounds generation from meat [44,45], probably due to different reasons for their complexity in the formation and also to eventual interactions [46]. The main source of VOCs is the lipid content. Most of the volatile compounds (acids, aldehydes, ketones, and alcohols) derived from lipid autoxidation and can themselves promote the formation of other components such as nitrogen and sulphur-containing compounds [44]. Moreover, *rigor-mortis* or *post-mortem* glycolytic ﬂuxes represented processes that are able to modify the volatile fraction of fresh meat [47].

The largest share of aromatic hydrocarbons is represented by the furan-2-pentyl-, which probably affected the statistical trend of the family. Other chemical compounds were much less represented; moreover, the detection of several molecules (toluene, benzene, benzene, 1,3-dimethyl-) would appear to be a consequence of their presence in animal feedstuffs and diet [48]. As suggested by several authors [49,50], the increase of aromatic hydrocarbons is mainly originated from lipid oxidation and has a relevant contribution to meat flavour, particularly a green bean/butter aroma is given by furan-2-pentyl-. The benzene is characterised with pleasant and distinct flavour, such as the sweeter 3-carene [51]. The toluene was characterised by fruity and sweet aroma as showed in goat meat by Madruga, et al. [52]. However, all of these VOCs are poorly represented in our study. Olivares, et al. [53] reported that toluene and ethylbenzene, the last one significantly decreased by ageing in our work, were most likely derived from amino acid degradation than lipid oxidation. The most represented aromatic hydrocarbon was the furan, 2-pentyl-, showing more than 85% of the total aromatic hydrocarbons, and it probably affects the statistical differences of the entire VOCs family. In similar studies that were conducted on horse meat vacuum aged, Maggiolino, et al. [54] and Tateo, Maggiolino, Domínguez, Lorenzo, Dinardo, Ceci, Marino, Della Malva, Bragaglio, and De Palo [25] observed that VOCs formation and trend changed depending on muscle considered. In fact, vacuum aging in *Semimembranosus* muscle only affected toluene, whereas in *Longissimus thoracis* muscle the vacuum aging modified the benzene and 3-carene content. But more interesting is the total absence of the furan, 2-penthyl- that is, instead, the most produced in *Longissimus thoracis* donkey meat. As said before, usually aromatic hydrocarbons derived from the lipid oxidation [49,50], but we observed a decreasing trend of total aromatic hydrocarbons VOCs. First of all, the lipid oxidation would be slightly depressed under vacuum aging [45], probably also for the low permeability of the film, and this can explain why aromatic hydrocarbons did not increase during the aging process. The results reported about oxidation products, as TBARs and hydroperoxides, strengthened this hypothesis. They remain constant during the aging period, as reported in horse meat [25], confirming that probably lipid oxidation is slightly depressed for both the short period and the packaging type [45,55], also considering the film low permeability. 

The total amount of aldehydes was not significantly affected by ageing. On the contrary, the values of many aldehydes individually changed, following a heterogeneous trend. First of all, we must consider the limited number of animals that are involved in the trial. This aspect may have influenced the large variability of results with no statistical differences. However, the hexanal that represented about the 85% of the total aldehydes and also the most abundant VOC generate did not show any change during the vacuum aging. This result agrees with studies that were conducted on horse meat vacuum packaged [25,54], but also with other studies conducted on same species (horse) in different storage conditions [56] and in different species and storage conditions as beef [57], pork [58], and lamb [59], in which researchers reported that hexanal is the most abundant VOC. Hexanal, which gives the meaty, grassy, and fatty odours to the meat, as the most of aldehydes, derived from phospholipids and polyunsaturated fatty acids oxidation [46,60,61], and the constant trend seems to validate the hypothesis of a reduced oxidative activity in meat vacuum stored. The authors reported a positive correlation between aldehydes formation and degree of oxidation, particularly with TBARs [62], and the absence of variation of both oxidative parameters and aldehydes seems to consolidate this hypothesis.

Linear hydrocarbons identified are the most numerous, although some authors pointed out their unimportant role in meat flavour [49], but not the most abundant, differently from that found by some authors in beef meat [45]. As observed in horse meat [25,54], the heptane,2,2,4,6,6-pentamethyl- and the undecane are the most abundant, representing around 20% and 10% of total linear hydrocarbons in cooked donkey meat, respectively. Although it seems that linear hydrocarbons also derived by autoxidation processes, particularly of long-chain fatty acids, are principally formed after lipids thermal homolyses [63], and therefore after heat treatments, as cooking. Only two linear hydrocarbons showed changes during aging time, pentane, and 2-octene, together representing less than 1% of total linear hydrocarbons, but the total hydrocarbons did not modify during the whole display. Our results can confirm the idea that oxidative processes are strongly reduced, probably due to the low oxygen permeability of the used film.

This study highlighted significant differences in three ketones: 2,3-pentanedione; 2-hexanone; and, 2-methyl-3-octanone, representing around 2% of the total ketones. In fact, the total ketones did not change during the aging process. Ketones are considered very important in giving flavour on cooked meat, in fact it is reported that are responsible of sweet, buttery, spicy, ethereal, caramel, cheese and fatty notes [49,54,64]. The main substrate responsible of their formation is the intramuscular fat, deriving from fatty acids oxidation, and their formation and production are positively correlated with intramuscular fat content [65]. However, this is not the only route of formation, because they can also derive from Maillard compounds [66] and from lipolytic activity and alkane degradation by microbial metabolism (β-oxidation) [67,68]. The very probable lack of oxidative processes on intramuscular fat and fatty acids and, consequently, the probable inactivation of microbial activity and the equal cooking method adopted, can easily explain the absence of variation of total ketones compounds during the aging time. However, is really interesting to observe that ketones formed in donkey meat are more abundant than those that are reported in horse meat vacuum packed [25,54], more than triple, since the acetoin was the most abundant ketones and about four times more than those found in vacuum packed horse meat.

The total alcohols showed a constant trend, and 1-hexanol was the most abundant. Although other authors observed in horse meat that this compound is the most abundant [25,54], they observed a different trend during the vacuum aging, showing increasing values. These compounds also derive from lipid oxidation, particularly from the degradation of oleic and linoleic acid [69], and their liberation depends on thermal treatment [70]. The absence of variation can be due to a probable inhibition of the oxidation processes during the vacuum aging. These compounds seem to give flavour described as resin, flower, and green aroma [51,56], but they also have a high odour threshold limit, so they are often classified as not important flavour contributors to meat [70].

Carboxylic acids and sulphur compounds did not show changes and were relatively poor generated, unlike what was observed for nitrogen compounds that tend to increase during the vacuum aging. Nitrogen compounds derived by the interaction of sulphur-free amino acids with sugars lead to the formation of these compounds, like e.g., pyrazines [71]. These compounds are particularly important, because of their lower limit aroma threshold and the garlic and onion aroma that they can give to meat. Our results agree with those observed in beef meat during the vacuum aging [64], since the authors noticed an increase in nitrogen compounds, justifying it with a plausible release of free amino acids during the aging process that enhance, in turn, Maillard reactions. This agrees with the protein carbonyls trend that is the result of protein oxidation, which, in turn, is responsible for the greater availability of free amino acids.

Similar to that reported in horse meat [25], antioxidant enzymes activity tend to increase during aging process. Superoxide dismutase, catalase and glutathione peroxidase represented the *in vivo* cell defence system against oxidative damage [72]. They lose their activity after slaughtering [73,74] due to denaturation processes and hydrolysis carried out by intracellular proteinases, and usually are characterised by a decreasing trend during aging, also for a redistribution of these enzymes between cellular compartments due to the rupture of cell walls [75,76]. In this regard, Tateo, Maggiolino, Domínguez, Lorenzo, Dinardo, Ceci, Marino, Della Malva, Bragaglio and De Palo [25] noticed that this result can be explained by the kind of measurement that is used for quantifying these enzymes in meat. It is expressed as quantity on mg of proteins and the protein degradation, that lead more free amino acids release, lead also to a reduction of the concentration measurement substrate, as result giving a higher concentration, but not a higher real enzyme availability.

It seems that sensory evaluation is poorly affected by vacuum aging, although there is a positive evaluation of the product at all aging days considered. Although aldehydes did not change during the aging process, their concentration is particularly high, more than that observed by other authors in different muscle in horse meat [25,54]. Their impact is significant on meat aroma [77], probably due to their low threshold odour [78], and this can justify the good values that were registered for meaty odour and overall licking. The increasing evaluation of sweetness could assert the idea that there is a major sugar components availability who themselves become available as a substrate, together with free amino acids, for the formation of nitrogen compounds.

## 5. Conclusions

The results of this study demonstrated that the vacuum aging of donkey meat poor affect the volatile compounds, although it is able to limit all oxidative processes, and so the formation of all VOCs that derive from lipid oxidation. It is also true that the meat samples are limited in the number (only 10), and this may have affected our results. However, although they did not change, aldehydes are particularly present, and nitrogen compounds, total aromatic hydrocarbons, and total carboxylic acids varied during aging influencing the sensory evaluation. Donkey meat vacuum aged increased sweetness, meaty odor and overall licking perceived by consumers. Protein oxidation and/or degradation happened and increased during aging, and this is indirectly observable by the protein carbonyl formation and the nitrogen compounds release that increased during aging. Enzyme activity increased with vacuum aging, which suggested their potential effect on meat oxidative processes after opening and meat shelf life. However, this study represents the first approach to donkey meat aging with vacuum packaging, giving us important and innovative results.

## Figures and Tables

**Table 1:** Effect of aging time in aromatic hydrocarbons and aldehydes content, expressed as quantifier area units (AU × 10^3^/g), of donkey meat steaks aged for 14 days under vacuum conditions (*n* = 10 samples for each aging time).

			Ageing Time (Days)						
Volatile Compound	*m*/*z*	LRI	1	3	6	9	14	SEM	*p*-Value
Furan, 3-methyl-	82	582	34.46	27.11	19.04	29.47	21.23	2.20	0.219
Benzene	78	650	122.13	122.00	68.75	80.10	86.78	9.63	0.262
Furan, 2,5-dihydro-	41	670	35.15	25.35	16.94	22.09	21.62	2.11	0.118
Furan, 2-ethyl-	81	703	338.52	287.36	119.13	156.62	178.18	27.25	0.054
Toluene	92	804	121.07	80.29	116.04	141.66	119.53	9.82	0.329
Ethylbenzene	91	917	561.00 ^a^	222.73 ^b^	248.60 ^b^	231.58 ^b^	207.55 ^b^	37.81	0.019
Benzene, 1,3-dimethyl-	106	926	585.07	446.28	501.35	458.53	484.48	36.24	0.821
2-n-Butyl furan	81	944	324.98	290.61	154.92	178.15	238.87	24.02	0.135
p-Xylene	106	958	221.17 ^a^	121.76 ^b^	128.72 ^a,b^	116.05 ^b^	120.83 ^b^	11.49	0.024
3-Carene	136	983	109.63	89.09	126.93	109.03	102.17	8.58	0.718
Furan, 2,3-dihydro-3-methyl-	81	984	94.92	46.31	102.23	84.43	58.42	15.74	0.765
Furan, 2-pentyl-	81	1043	10,213.41 ^a^	8082.13 ^a,b^	3462.15 ^b^	5171.65 ^a,b^	5534.13 ^a,b^	730.69	0.032
Total Aromatic Hydrocarbons			12,375.25 ^a^	9838.03 ^a,b^	5064.81 ^b^	6779.35 ^a,b^	7173.80 ^a,b^	813.14	0.046
Propanal	58	526	580.18	809.05	596.61	651.52	870.97	50.04	0.258
Propanal, 2-methyl-	72	557	24.63 ^a,b^	15.96 ^a^	19.06 ^b^	34.31 ^b^	27.65 ^b^	1.95	0.010
Butanal	72	584	56.44 ^a,b^	60.67 ^a,b^	48.67 ^a^	52.48 ^a,b^	78.13 ^b^	3.31	0.042
Butanal, 3-methyl-	58	659	30.03 ^a^	34.61 ^a^	28.29 ^a^	79.21 ^b^	55.48 ^a,b^	5.62	0.011
Butanal, 2-methyl-	57	671	45.14	62.95	45.45	108.90	87.94	8.64	0.087
Pentanal	57	728	2402.89	2113.87	2207.51	2285.09	2858.49	1.89	0.335
Hexanal	56	865	34,161.17	33,955.98	36,215.04	35,474.08	38,958.15	3.31	0.732
Furfural	95	933	39.37	23.43	29.28	53.39	24.28	5.62	0.111
2-Hexenal, (E)-	41	943	31.37	39.90	41.90	40.58	43.99	8.64	0.288
Heptanal	70	974	1012.65	1437.75	1090.78	1258.64	1436.41	122.08	0.303
Benzaldehyde	106	1045	248.02 ^a,b^	258.45 ^a,b^	221.45 ^a^	335.76 ^a,b^	481.34 ^b^	1231.03	0.031
Octanal	57	1086	352.34	513.19	408.50	413.44	511.60	4.18	0.506
5-Ethylcyclopent-1-enecarboxaldehyde	124	1099	52.37 ^a,b^	59.46 ^a,b^	35.52 a	48.00 ^a,b^	66.62 ^b^	1.76	0.025
Benzeneacetaldehyde	91	1119	29.02 ^a,b^	23.27 ^a^	38.55 ^a,b^	85.76 ^b^	58.54 ^a,b^	77.33	0.030
2-Octena(E)-	70	1123	73.42 ^a^	69.16 ^a^	45.44 ^a,b^	38.01 ^b^	64.86 ^a,b^	29.67	0.005
Nonanal	57	1148	516.48	813.20	653.26	641.12	718.82	33.44	0.179
Benzaldehyde, 3-ethyl-	134	1209	24.25 ^a,b^	38.46 b	17.66 ^a^	34.19 ^a,b^	25.62 ^a,b^	2.39	0.023
2,4-Decadienal	81	1315	27.63	29.43	26.99	21.60	29.85	7.32	0.689
Total Aldehydes			40,132.94	40,321.95	41,764.45	41,637.05	46,387.71	3.85	0.687

Different letters in the same line show statistical differences (^a,b^: *p* < 0.01); SEM: standard error of mean; *m*/*z*: Quantifier ion; LRI: Lineal Retention Index calculated for DB-624 capillary column (J&W scientific: 30 m × 0.25 mm id, 1.4 µm film thickness) installed on a gas chromatograph equipped with a mass selective detector. LRI: linear retention index in agreement with literature for the same chromatographic column [25,34,43].

**Table 2:** Effect of aging time in the hydrocarbons content, expressed as quantifier area units (AU × 10^3^/g), of donkey meat steaks aged for 14 days under vacuum conditions (*n* = 10 samples for each aging time).

			Ageing Time (Days)						
Volatile Compound	*m*/*z*	LRI	1	3	6	9	14	SEM	*p*-Value
Pentane	43	500	119.92 ^a^	99.34 ^a,b^	69.29 ^b,c^	38.12 c	83.47 ^a,b^	6.51	<0.001
n-Hexane	69	600	154.90	135.22	124.46	128.71	90.79	7.10	0.084
Hexane, 2,2-dimethyl-	57	660	59.22	51.24	81.76	72.33	61.21	4.81	0.270
Isopropylcyclobutane	56	670	59.76	40.85	27.63	33.99	35.41	3.82	0.125
Heptane	71	700	145.35	154.56	168.56	65.65	121.28	12.27	0.059
Pentane, 2,3,4-trimethyl-	71	756	19.48	23.92	34.00	38.94	25.68	3.63	0.492
Heptane, 3,3,4-trimethyl-	71	756	24.50	29.34	43.01	50.93	28.61	4.94	0.440
Hexane, 2,2,4-trimethyl-	57	804	42.65	50.42	81.95	91.10	45.90	9.55	0.392
2-Heptene, 3-methyl-	70	817	54.92	68.88	102.49	109.74	54.79	9.14	0.178
Octane	85	800	938.35	709.11	778.72	342.71	569.13	66.36	0.065
2-Octene, (E)-	112	833	39.06 ^a,b^	45.17 ^a,b^	56.26 ^b^	21.97 ^a^	29.16 ^a,b^	3.82	0.032
Cyclohexane, 1,2-dimethyl- (cis/trans)	55	837	48.70	51.58	39.03	28.69	37.46	3.51	0.222
Heptane, 2,3-dimethyl-	43	847	22.54	24.65	41.26	34.94	21.01	3.85	0.420
4-Octene, (E)-	55	849	28.12	24.91	29.19	16.53	16.58	1.99	0.117
Bicyclo[2.2.2]octane	67	869	36.40	60.50	33.97	33.08	45.22	5.69	0.462
Octane, 2-methyl-	57	903	18.58	15.90	23.08	20.92	17.12	1.49	0.557
Heptane, 3-ethyl-	57	910	176.78	200.08	340.87	261.61	158.73	32.69	0.428
Nonane, 3,7-dimethyl-	57	920	81.04	98.91	182.68	140.14	76.77	16.96	0.254
Heptane, 2,2,4-trimethyl-	57	926	189.83	199.64	346.37	255.98	154.68	33.16	0.431
Heptane, 2-methyl-3-methylene-	57	935	25.76	37.88	50.57	40.64	31.65	4.71	0.606
Octane, 3,5-dimethyl-	57	940	183.17	187.43	306.95	226.50	150.97	28.76	0.520
3-Methyl-3-hexene	83	1042	53.76	57.27	90.26	64.45	47.09	8.09	0.536
2-Octene, 4-ethyl-, (E)-	69	982	152.48	179.33	263.42	177.96	160.62	24.90	0.690
Heptane, 3-ethyl-5-methylene-	70	989	256.88	304.39	469.75	359.75	281.88	42.93	0.596
3-Ethyl-3-methylheptane	57	992	83.30	86.41	129.37	96.89	73.42	11.47	0.632
Pentane, 3,3-dimethyl-	43	999	21.65	25.03	35.32	28.03	27.20	2.06	0.360
Undecane, 6,6-dimethyl-	57	1010	113.94	106.05	168.03	113.08	93.69	16.06	0.674
Nonane, 5-methylene-	56	1015	124.18	125.96	184.13	138.12	119.34	16.99	0.780
2-Nonene, 3-methyl-, (E)-	70	1026	272.98	329.03	454.00	343.21	304.51	42.60	0.773
Heptane, 2,2,4,6,6-pentamethyl-	57	1027	2097.53	2047.35	2887.36	2376.41	2075.11	243.39	0.814
Decane	57	1000	469.70	457.81	617.97	465.74	448.75	57.99	0.898
(Z)-4-Methyl-2-hexene	98	1060	139.74	137.23	178.97	150.10	125.24	15.12	0.861
2,2,4,4-Tetramethyloctane	57	1066	211.32	184.91	320.03	236.37	198.07	28.68	0.609
Undecane, 5,5-dimethyl-	57	1084	207.26	193.58	278.17	212.43	187.80	23.16	0.770
Dodecane, 2,6,10-trimethyl-	57	1092	163.67	134.96	189.02	154.10	111.87	13.95	0.513
Dodecane, 4-methyl-	43	1105	113.87	97.93	144.11	113.03	89.44	12.08	0.693
2-Decene, 3-methyl-, (Z)-	57	1110	82.78	79.19	103.54	75.12	76.97	9.20	0.889
Undecane	57	1100	1373.12	1104.57	1573.01	1321.27	1116.49	127.76	0.774
2-Undecene, 9-methyl-, (Z)-	57	1137	302.41	303.61	428.77	345.53	301.68	36.10	0.794
2-Acetyl-2-methyltetrahydrofuran	57	1147	35.28	34.23	44.74	34.45	38.20	3.39	0.872
4,4-Dipropylheptane	57	1161	14.31	16.74	20.38	14.75	15.59	1.02	0.388
Pentane, 3,3-diethyl-	57	1166	112.29	113.67	86.75	91.55	75.91	13.17	0.883
Dodecane, 2-methyl-6-propyl-	57	1173	183.67	157.97	237.76	206.75	144.99	19.34	0.582
2-Undecene, 3-methyl-, (E)-	70	1181	57.00	57.19	75.93	64.50	54.04	6.32	0.843
Dodecane	57	1200	639.62	545.87	778.83	693.49	526.25	60.26	0.677
Pentadecane, 6-methyl-	57	1223	54.10	51.31	69.59	60.52	48.79	5.45	0.784
Decane, 2,3,6-trimethyl-	57	1238	17.80	15.30	20.01	15.86	14.27	0.98	0.393
Tridecane	71	1300	157.67	148.36	187.06	156.25	121.01	13.75	0.710
Tridecane, 3-methyl-	85	1304	16.57	19.63	21.41	17.97	13.53	1.21	0.311
Tetradecane	57	1400	23.85	25.28	19.53	17.16	14.76	1.77	0.271
Total Linear Hydrocarbons			10,311.94	9379.77	12,983.62	10,142.36	8707.29	1004.34	0.746

Different letters in the same line show statistical differences (^a,b,c^: *p* < 0.01). SEM: standard error of mean; *m*/*z*: Quantifier ion; LRI: Lineal Retention Index calculated for DB-624 capillary column (J&W scientific: 30 m × 0.25 mm id, 1.4 µm film thickness) installed on a gas chromatograph equipped with a mass selective detector. LRI: linear retention index in agreement with literature for the same chromatographic column [25,34,43].

**Table 3:** Effect of aging time in the ketones content, expressed as quantifier area units (AU × 10^3^/g), of donkey meat steaks aged for 14 days under vacuum conditions (*n* = 10 samples for each aging time).

			Ageing Time (Days)						
Volatile Compound	*m*/*z*	LRI	1	3	6	9	14	SEM	*p*-Value
2,3-Butanedione	43	592	203.73	165.32	145.96	94.94	123.05	39.25	0.586
2-Butanone	72	596	61.77	47.36	69.32	103.30	56.65	2.36	0.096
2-Pentanone	86	720	19.67	16.61	16.78	14.93	16.86	1.90	0.568
3-Pentanone	57	736	77.94	86.39	87.04	46.35	83.31	1445.53	0.447
2,3-Pentanedione	100	739	76.16 ^a^	161.23 ^b^	137.75 ^a,b^	99.43 ^a,b^	149.03 ^a,b^	20.34	0.011
Acetoin	45	787	1735.75	1540.13	1392.70	790.07	436.27	7.06	0.149
2-Hexanone	58	863	52.48 ^a^	38.46 ^a,b^	19.55 ^b^	27.82 ^a,b^	26.74 ^a,b^	0.80	0.016
3-Heptanone	57	960	22.90	32.27	31.33	30.30	34.04	7.75	0.607
2-Heptanone	58	967	1055.89	1285.42	648.08	943.32	1023.91	9.22	0.239
4-Cyclopentene-1,3-dione	96	1003	39.23	71.76	65.49	56.37	50.62	545.25	0.175
4-Hexen-3-one, 5-methyl-	83	1047	54.19	39.88	25.56	29.30	34.18	3.23	0.148
Butyrolactone	42	1049	87.11	61.90	78.59	84.56	79.82	2.10	0.346
2(5H)-Furanone	55	1053	259.73	129.48	80.36	265.89	50.97	91.23	0.282
3-Octen-2-one	55	1111	48.42	70.55	37.78	42.52	73.75	4.79	0.083
3-Octanone, 2-methyl-	43	1141	32.40 ^a^	62.72 ^a,b^	45.55 ^a,b^	34.68 ^a,b^	64.55 ^b^	3.62	0.012
2-Nonanone, 3-(hydroxymethyl)-	43	1146	15.27	17.35	19.34	22.43	17.61	4.39	0.404
Total Ketones			4210.12	2888.86	1347.94	2342.08	913.27	38.21	0.080

Different letters in the same line show statistical differences (^a,b^: *p* < 0.01). SEM: standard error of mean; *m*/*z*: Quantifier ion; LRI: Lineal Retention Index calculated for DB-624 capillary column (J&W scientific: 30 m × 0.25 mm id, 1.4 µm film thickness) installed on a gas chromatograph equipped with a mass selective detector. LRI: linear retention index in agreement with literature for the same chromatographic column [25,34,43].

**Table 4:** Effect of aging time in the alcohols, carboxylic acids, nitrogen compounds, and sulphur compounds content, expressed as quantifier area units (AU × 10^3^/g), of donkey meat steaks aged for 14 days under vacuum conditions (*n* = 10 samples for each aging time).

			Ageing Time (Days)						
Volatile Compound	*m*/*z*	LRI	1	3	6	9	14	SEM	*p*-Value
Cyclobutanol	44	504	143.69	169.85	147.22	138.39	143.89	4.76	0.957
1-Pentanol	55	847	2755.10	2733.92	1463.86	2160.24	1930.46	3.98	0.547
1-Hexanol	56	959	2130.13	2067.04	2218.5	1341.74	2341.82	1.12	0.729
1-Heptanol	70	1046	365.67	248.69	59.14	116.04	92.70	680.64	0.160
1-Octen-3-ol	57	1051	1418.47	1662.70	814.05	1001.59	1393.13	14.12	0.121
n-Tridecan-1-ol	55	1073	182.45	247.07	242.18	225.81	244.91	274.05	0.771
1-Heptanol, 2,4-diethyl-	69	1085	133.83	129.36	230.15	146.55	137.41	2488.83	0.601
1-Decanol	70	1027	21.60	25.42	34.15	26.52	20.61	42.70	0.488
1-Tetradecanol	68	1225	25.66	28.03	33.32	28.62	22.28	118.82	0.764
1-Decanol, 2-hexyl-	97	1241	21.03	22.45	28.21	24.67	16.65	16.25	0.491
1-Butanol, 2-methyl-	57	1243	18.14	16.15	19.77	17.15	17.39	22.01	0.943
Total Alcohols			7655.53	6345.87	3301.09	5243.44	4372.90	822.52	0.237
Butanoic acid	60	918	45.42	70.17	57.60	83.42	106.55	1.994	0.336
Hexanoic acid	60	1088	73.60	83.20	70.53	49.83	102.80	1.357	0.172
Formica cid, octyl ester	56	1133	47.48	110.11	92.78	122.76	108.28	1.44	0.282
Total Carboxylic Acids			169.82 ^a^	217.63 ^a,b^	208.69 ^ab^	219.33 ^a,b^	317.62 ^b^	2797.109	0.003
Diazene, dimethyl-	15	532	213.87	202.38	140.06	237.28	245.35	9.366	0.487
Pyrazine, methyl-	94	893	79.65 ^a^	57.10 ^a^	100.68 ^a^	163.72 ^b^	165.78 ^b^	6.782	0.019
2-Propen-1-amine	56	916	46.69	105.47	137.19	78.33	59.53	10.758	0.523
Pyrazine, 2,5-dimethyl-	42	982	223.78 ^a^	168.44 ^a^	277.77 ^a^	475.65 ^b^	668.82 ^b^	14.637	0.009
Pyrazine, trimethyl-	122	1064	128.90 ^a^	93.50 ^a^	154.74 ^a^	277.12 ^b^	293.64 ^b^	19.326	<0.001
Total Nitrogen Compound			606.07 ^a^	626.90 ^a^	810.43 a	1232.10 ^b^	1633.13 ^b^	11.795	0.004
Dimethylsulfide	62	534	26.63	21.63	15.03	24.71	17.47	17.208	0.558
Carbondisulfide	76	533	119.65	117.40	94.24	132.27	133.34	33.188	0.540
Total Sulfur Compound			136.65	146.63	123.96	144.38	157.85	16.872	0.761

Different letters in the same line show statistical differences (^a,b^: *p* < 0.01). SEM: standard error of mean; *m*/*z*: Quantifier ion; LRI: Lineal Retention Index calculated for DB-624 capillary column (J&W scientific: 30 m × 0.25 mm id, 1.4 µm film thickness) installed on a gas chromatograph equipped with a mass selective detector. LRI: linear retention index in agreement with literature for the same chromatographic column [25,34,43].

**Table 5:** Effect of aging time on Thiobarbituric Acid Reactive Substances (TBARs), hydroperoxides, protein carbonyls, superoxide dismutase, catalase, and glutathione peroxidase of donkey meat steaks aged for 14 days under vacuum conditions (*n* = 10 samples for each aging time).

	Ageing Time (Days)						
Item	1	3	6	9	14	SEM	*p*-Value
TBARs (mg MDA/kg of meat)	0.83	0.83	1.08	1.29	1.19	0.12	0.0832
Hydroperoxides (mmol/g of meat)	0.53	0.71	0.58	0.70	0.73	0.05	0.4551
Protein carbonyls (mmol DNPH/mg protein)	2.06 ^a^	2.80 ^b^	2.73 ^b^	2.73 ^b^	2.68 ^b^	0.29	0.2902
Superoxide dismutase (U/mg protein)	8.48 ^a^	12.28 ^b^	18.57 ^c^	23.57 ^d^	25.05 ^e^	0.10	<0.0001
Catalase (U/mg protein)	3.23 ^a^	4.41 ^b^	5.36 ^c^	6.31 ^d^	7.13 ^e^	0.05	<0.0001
Glutathione peroxidase (µmol NADPH ox/mg protein)	6.25 ^a^	7.71 ^b^	9.20 ^c^	11.59 ^d^	13.44 ^e^	0.04	<0.0001

Different letters in the same line show statistical differences (^a,b,c,d,e^: *p* < 0.01). SEM: standard error of mean.

**Table 6:** Effect of aging time on sensory panel evaluation foal meat steaks aged for 14 days under vacuum conditions (*n* = 20 samples for each aging time).

Item	Aging Time (Days)					SEM	*p*-Value
	1d	3d	6d	9d	14d		
Tenderness	6.88	7.05	7.21	6.99	7.06	0.04	0.3220
Juiciness	6.95	7.15	7.02	6.89	6.59	0.09	0.2789
Sweetness	7.45 ^a^	7.41	7.88	7.95	8.12 ^b^	0.18	0.0179
Unpleasant taste	4.38	4.51	4.75	4.66	4.29	0.36	0.6338
Unpleasant odor	5.56	5.71	5.78	5.66	5.32	0.19	0.5538
Meaty odor	6.89 ^a^	6.95	6.87	6.99	7.58 ^b^	0.21	0.0121
Overall liking	7.12 ^a^	7.22	7.15	7.36	7.81 ^b^	0.09	0.0207

Different letters in the same line show statistical differences (^a,b^: *p* < 0.05). SEM: standard error of mean.
